# Glutamate as a potential “survival factor” in an in vitro model of neuronal hypoxia/reoxygenation injury: leading role of the Na^+^/Ca^2+^ exchanger

**DOI:** 10.1038/s41419-018-0784-6

**Published:** 2018-06-28

**Authors:** Silvia Piccirillo, Pasqualina Castaldo, Maria Loredana Macrì, Salvatore Amoroso, Simona Magi

**Affiliations:** Department of Biomedical Sciences and Public Health, School of Medicine, University “Politecnica delle Marche”, Via Tronto 10/A, 60126 Ancona, Italy

## Abstract

In brain ischemia, reduction in oxygen and substrates affects mitochondrial respiratory chain and aerobic metabolism, culminating in ATP production impairment, ionic imbalance, and cell death. The restoration of blood flow and reoxygenation are frequently associated with exacerbation of tissue injury, giving rise to ischemia/reperfusion (I/R) injury. In this setting, the imbalance of brain bioenergetics induces important metabolic adaptations, including utilization of alternative energy sources, such as glutamate. Although glutamate has long been considered as a neurotoxin, it can also be used as intermediary metabolite for ATP synthesis, and both the Na^+^/Ca^2+^ exchanger (NCX) and the Na^+^-dependent excitatory amino-acid transporters (EAATs) are essential in this pathway. Here we analyzed the role of NCX in the potential of glutamate to improve metabolism and survival of neuronal cells subjected to hypoxia/reoxygenation (H/R). In SH-SY5Y neuroblastoma cells differentiated into a neuron-like state, H/R produced a significant cell damage, a decrease in ATP cellular content, and intracellular Ca^2+^ alterations. Exposure to glutamate at the onset of the reoxygenation phase attenuated H/R-induced cell damage and evoked a significant raise in intracellular ATP levels. Furthermore, we found that in H/R cells NCX reverse-mode activity was reduced, and that glutamate limited such reduction. All the effects induced by glutamate supplementation were lost when cells were transfected with small interfering RNA against NCX1 and EAAT3, suggesting the need of a specific functional interplay between these proteins for glutamate-induced protection. Collectively, our results revealed the potential beneficial effect of glutamate in an in vitro model of H/R injury and focused on the essential role exerted by NCX1. Although preliminary, these findings could be a starting point to further investigate in in vivo systems such protective effect in ischemic settings, shedding a new light on the classical view of glutamate as detrimental factor.

## Introduction

Brain ischemia is a pathological condition characterized by blood supply and oxygen deprivation, which alter energy metabolism and lead to cell death^1^. Brain energy demands are high, requiring a continuous supply of oxygen and glucose. Interruption of blood flow generates a mismatch between energy consumption and energy production, leading to complex energy-dependent cellular damage. In particular, the oxygen deficit affects oxidative phosphorylation and ATP production, leading to dysfunction of ATP-dependent ion transport pumps and to ionic imbalances^1, 2^. An exacerbation of the tissue damage frequently occurs upon restoration of blood flow and the concomitant reoxygenation, giving rise to the so-called “ischemia/reperfusion (I/R) injury”^3, 4^. In this setting, the cell loss can be blocked during the first few hours of reperfusion^4^, especially in the penumbra, the area in which energy metabolism is more moderately affected. Therefore, prompt interventions during this time window can avoid the development of irreversible damage in neurons and, in this regard, the stimulation of oxidative metabolism has recently gained much attention^5, 6^. The imbalance in brain bioenergetics produced by I/R induces important metabolic adaptations, including the utilization of alternative energy sources, such as glutamate, glutamine, and gamma-aminobutyric acid (GABA)^7, 8^. Among them, glutamate is an abundant substrate with the potential to be used by several fuel-providing mechanisms to rescue the cell from energy impairment, through its ability to link carbohydrate and amino-acid metabolism via the tricarboxylic acid (TCA) cycle^9^. Indeed, under energy-depleted conditions, the stimulation of glutamate conversion to alpha-ketoglutarate is able to restore ATP levels by supporting continuous TCA cycling^5^. By using different cell models (neurons, astrocytes, and cardiac cells)^10, 11^, we previously demonstrated that under physiological conditions, glutamate increases the ATP cellular content through a mechanism that involves two main cellular transporters: the Na^+^/Ca^2+^ exchanger (NCX) and the Na^+^-dependent excitatory amino-acid transporters (EAATs). NCX is a key player in controlling ionic homeostasis at both the plasma membrane and mitochondrial levels^12, 13^, as it can facilitate both Ca^2+^ and Na^+^ flow in a bidirectional way^14, 15^. NCX belongs to a multigene family comprising three isoforms named NCX1, NCX2, and NCX3, which are differentially distributed through the body. NCX1 is ubiquitously expressed in all tissues, NCX2 is mainly restricted to the brain, and NCX3 is expressed exclusively in the brain and the skeletal muscles^16^. EAATs are secondary active, electrogenic transport systems that couple the accumulation of glutamate in the cytoplasm to downhill movement of cotransported ions along their concentration gradient^17^. We reported a physical and functional interaction between NCX1 and a member of the EAAT family, EAAT3 (Excitatory Amino-Acid Carrier 1—EAAC1—in rats^18^), at both the plasma membrane and mitochondrial levels^10, 11^. These proteins cooperate to promote glutamate entry into the cytoplasm and then into mitochondria, where glutamate serves as a fuel for ATP synthesis^10, 11^. On the basis of these findings, the present study aimed (1) to investigate whether glutamate supplementation during the reoxygenation phase could ameliorate the energy state of the cell and protect against hypoxia/reoxygenation (H/R) injury and (2) to explore the involvement of NCX1 and EAAT3 in this metabolic response. As an in vitro model we used SH-SY5Y human neuroblastoma cells differentiated with retinoic acid (RA) to a neuron-like state.

## Results

### Protective effect of glutamate on H/R-induced cell injury: involvement of NCX1

**Fig. 1:**
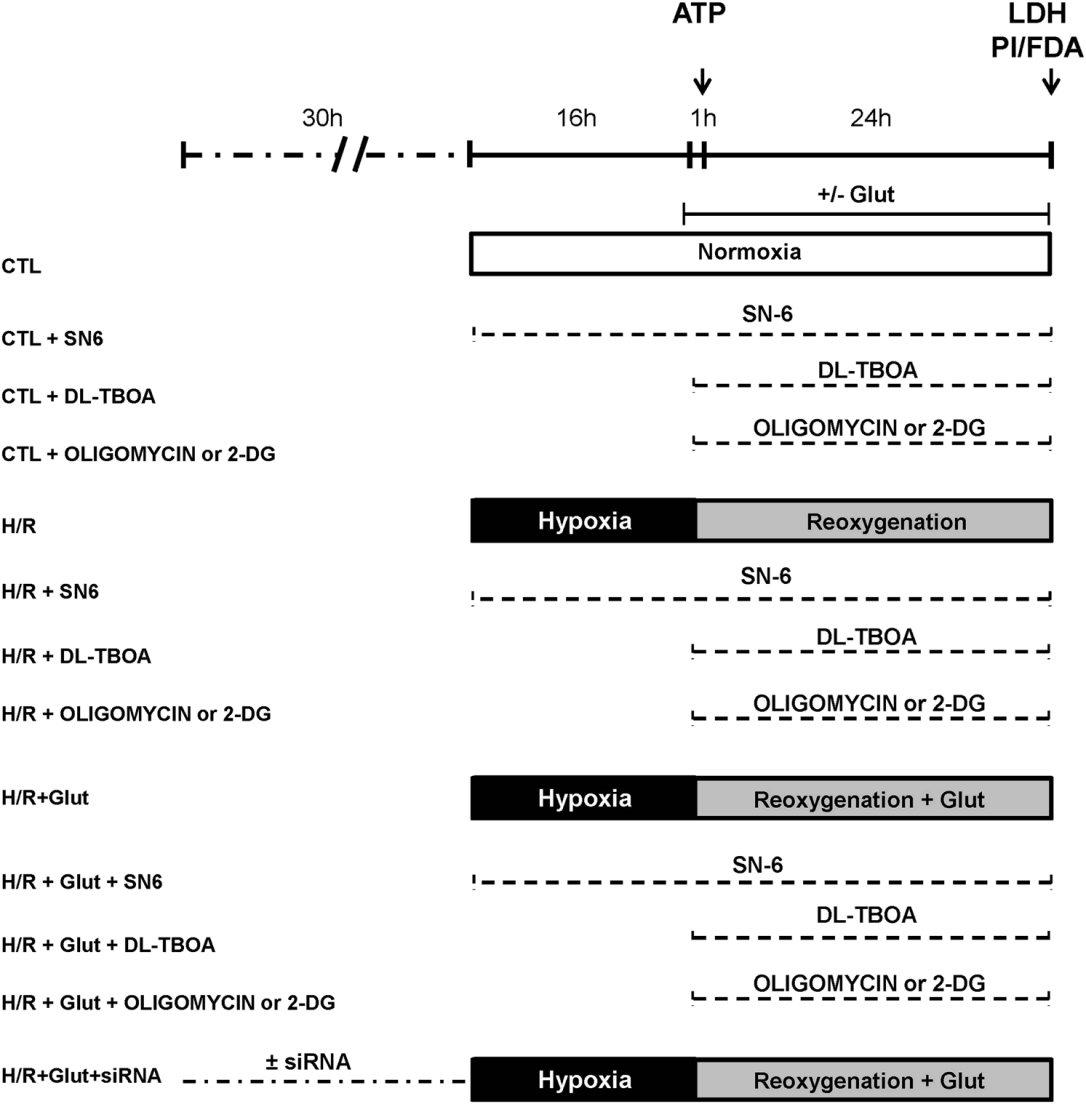
Timeline of the experimental protocol (H/R) in RA-differentiated SH-SY5Y cells. Control groups were incubated under normoxic conditions at 37 °C for the entire protocol. Glutamate (0.5 mM) and drugs (DL-TBOA, oligomycin, and 2-DG) were administrated during the reoxygenation phase. SN-6 was administrated during the entire H/R protocol. Cell viability (assessed by extracellular LDH measurement) was evaluated at the end of the protocol; ATP levels were evaluated after the first hour of reoxygenation. CTL control, H/R hypoxia/reoxygenation, Glut glutamate, 2-DG 2-deoxyglucose

**Fig. 2:**
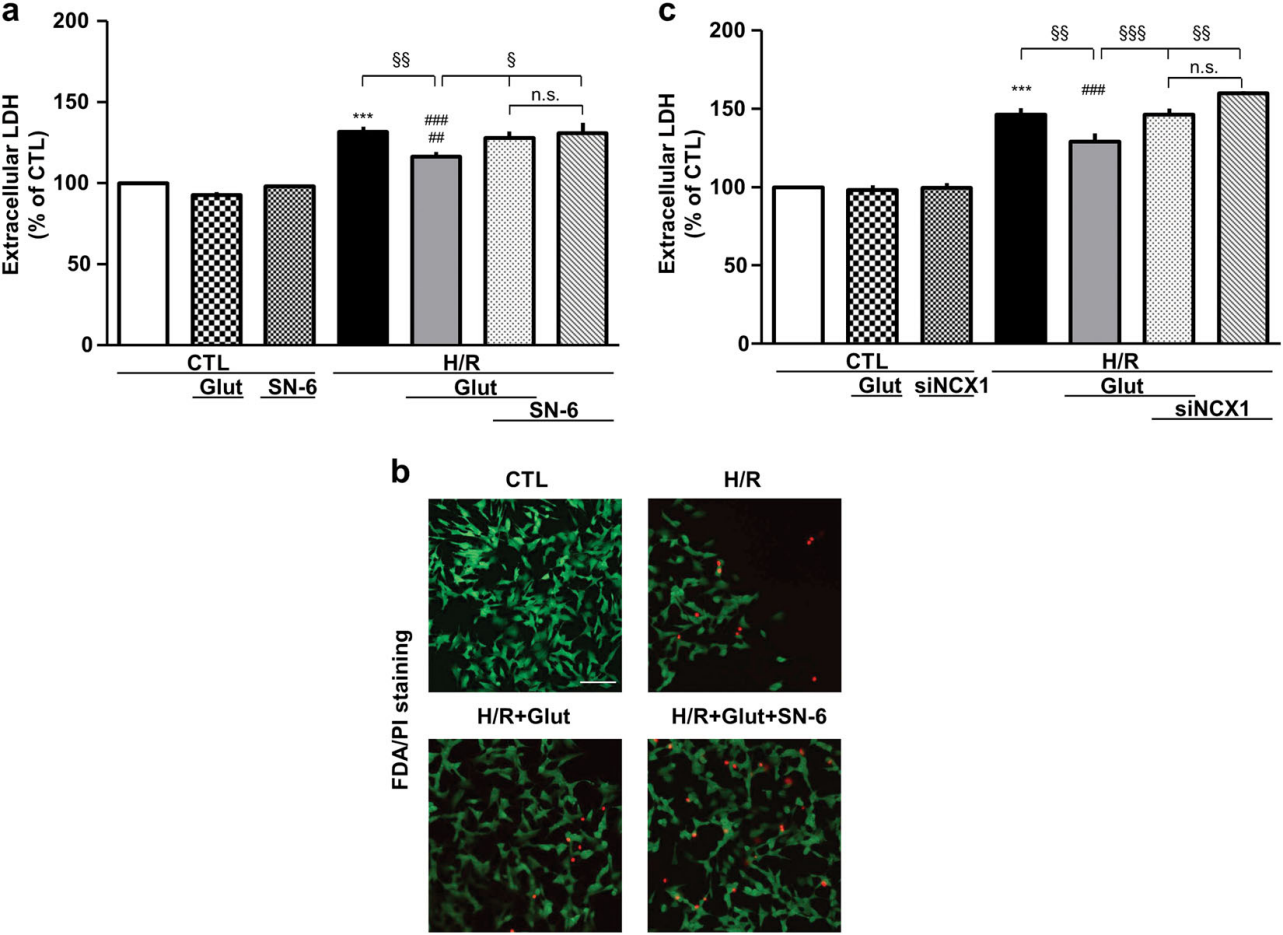
Glutamate-induced protection against H/R injury is prevented by NCX1 inhibition. Extracellular LDH activity measured at the end of the H/R protocol (16 h of hypoxia followed by 24 h of reoxygenation) under different experimental conditions. In each experiment, LDH release was expressed as percentage of the control. **a** Glutamate (0.5 mM) was added during the reoxygenation phase, alone or in combination with 1 μM SN-6, according to the time schedule showed in Fig. 1. Each column represents the mean ± S.E.M. of almost five independent experiments performed in triplicate. Where unseen, error bars overlap with the histogram outline. Differences among means were assessed by one-way ANOVA followed by Dunnet’s post hoc test. *F*(6, 70) = 29.57. ***p < 0.0001 vs control groups; ^###^*p* < 0.0001 vs CTL, CTL+Glut; ^##^*p* < 0.001 vs CTL+SN-6; ^§§^*p* < 0.001, ^§^*p* < 0.01 vs the indicated groups. The H/R group was not significantly different from the H/R + SN-6 + Glut and H/R + SN-6 groups. **b** Analysis of cell survival by FDA/PI staining. Images are representative of three independent experiments. Scale bar 50 µm. **c** Silencing of NCX1 was performed 30 h before H/R challenge. Glutamate was added at the onset of the reoxygenation phase. Each column represents the mean ± S.E.M. of almost five independent experiments performed in triplicate. Differences among means were assessed by one-way ANOVA followed by Dunnet’s post hoc test. *F*(6, 29) = 44.76. ***p < 0.0001 vs control groups; ^§§§^*p* < 0.0001, ^§§^*p* < 0.001 vs the indicated groups. The H/R group was not significantly different from the H/R + siNCX1 + Glut and H/R + siNCX1 groups. There was no statistically significant difference between the control groups. CTL control, H/R hypoxia/reoxygenation, Glut glutamate, siNCX1 siRNA for NCX1, n.s. not significant

We initially developed an in vitro model of neuronal H/R by using RA-differentiated SH-SY5Y cells. When cells were subjected to 16 h of hypoxia (H) followed by 24 h of reoxygenation (R) (Fig. 1), we found that cell damage was significantly higher compared to that of normoxic control (Fig. 2a, b). To study whether glutamate could attenuate H/R injury, cells were treated with glutamate (0.5 mM) at the onset of the reoxygenation phase (Fig. 1). We found that such supplementation significantly attenuated H/R damage (approximately 50% compared to H/R cells), without any sign of toxicity under normoxic conditions (Fig. 2). To explore the specific contribution of NCX1, we first used a pharmacological approach. When cells were exposed to the selective NCX inhibitor 2-[[4-[(4Nitrophenyl) methoxy] phenyl] methyl]-4-thiazolidinecarboxylic acid ethyl ester (SN-6, 1 µM)^19, 20^, glutamate was wholly ineffective in protecting cells from H/R injury (Fig. 2a, b). SN-6 per se had no effect on cell viability (Figs. 1 and 2a). To specifically assess the role of NCX1, we used an RNA interference (RNAi)-mediated approach to silence either NCX1 or NCX3 expression (Supplementary Fig. 1). Silencing of NCX2 isoform was not performed because under our culture conditions SH-SY5Y cells do not express NCX2 protein, neither when undifferentiated^21^ nor after RA treatment (data not shown). As shown in Fig. 2c, NCX1 silencing significantly prevented cell injury mitigation induced by glutamate. Of note, we found that NCX3 was not involved in this phenomenon (Supplementary Fig. 2), supporting the hypothesis that NCX1 is the specific isoform requested for glutamate to exert its beneficial effect.

### Protective effect of glutamate on H/R-induced cell injury: involvement of EAAT3

**Fig. 3:**
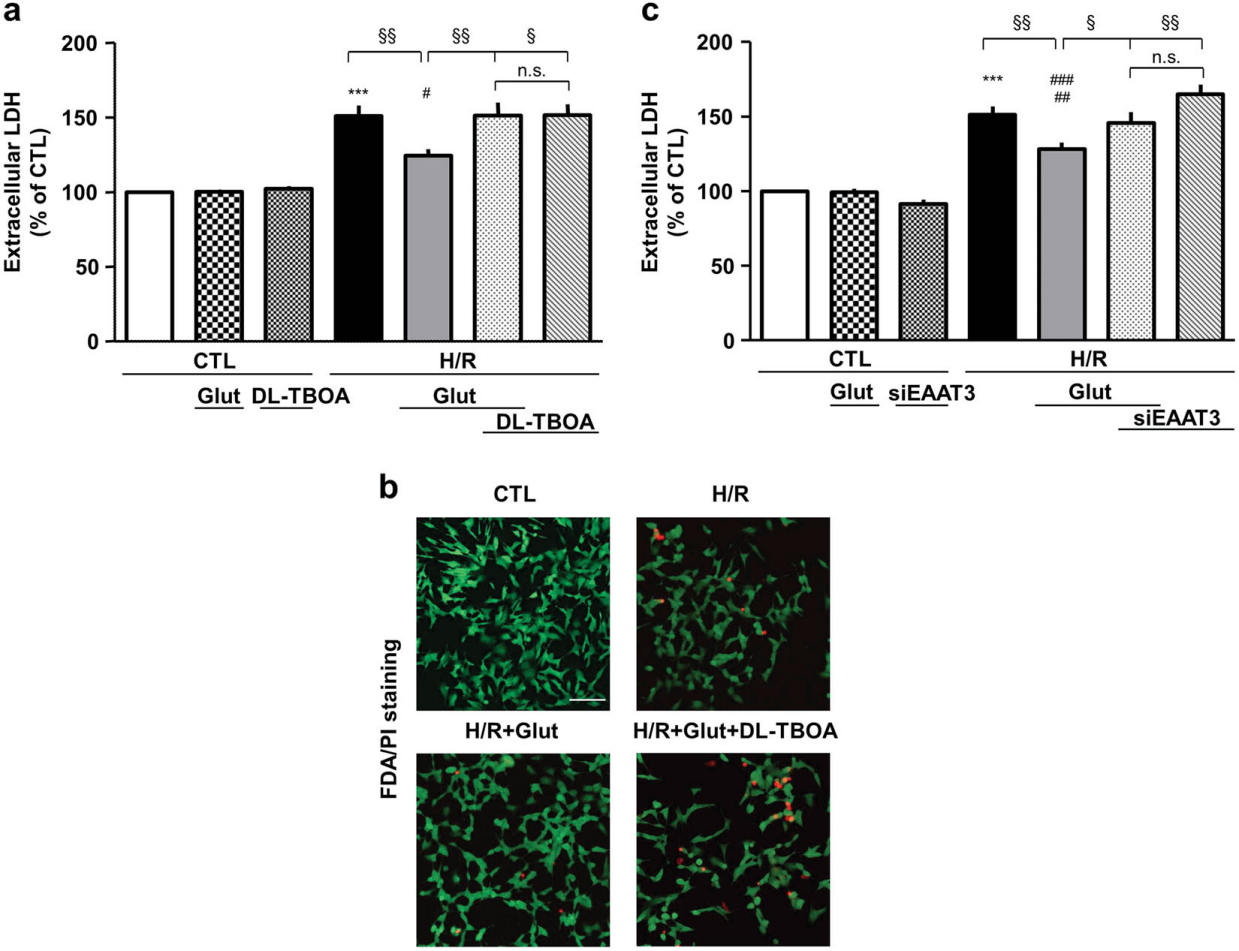
Glutamate-induced protection against H/R injury is prevented by EAAT3 inhibition. Extracellular LDH activity measured at the end of the H/R protocol (16 h of hypoxia followed by 24 h of reoxygenation) under different experimental conditions. In each experiment, LDH release was expressed as percentage of the control. **a** Glutamate (0.5 mM) was added during the reoxygenation phase, alone or in combination with 300 μM DL-TBOA, according to the time schedule showed in Fig. 1. Each column represents the mean ± S.E.M. of almost eight independent experiments performed in triplicate. Where unseen, error bars overlap with the histogram outline. Differences among means were assessed by one-way ANOVA followed by Dunnet’s post hoc test. *F*(6, 42) = 18.38. ***p < 0.0001 vs control groups; #*p* < 0.01 vs CTL and CTL+Glut; ^§§^*p* < 0.001, ^§^*p* < 0.01 vs the indicated groups. The H/R group was not significantly different from the H/R + DL-TBOA + Glut and H/R + DL-TBOA groups. **b** Analysis of RA-differentiated SH-SY5Y cell survival by FDA/PI staining. Images are representative of three independent experiments. Scale bar 50 µm. **c** Silencing of EAAT3 was performed 30 h before the H/R challenge. Glutamate was added at the onset of the reoxygenation phase. Each column represents the mean ± S.E.M. of almost six independent experiments performed in triplicate. Differences among means were assessed by one-way ANOVA followed by Dunnet’s post hoc test. *F*(6, 53) = 25.33. ***p < 0.0001 vs control groups; ^###^*p* < 0.0001 vs CTL and CTL + siEAAT3; ^##^*p* < 0.001 vs CTL + Glut; ^§§^*p* < 0.001, ^§^*p* < 0.01 vs the indicated groups. The H/R group was not significantly different from the H/R + siEAAT3 + Glut and H/R + siEAAT3 groups. There was no statistically significant difference between the control groups. CTL control, H/R hypoxia/reoxygenation, Glut glutamate, siEAAT3 siRNA for EAAT3, n.s. not significant

It is well established that EAATs mediate the high-affinity uptake of glutamate. We demonstrated that under both physiological and pathological conditions^10, 11, 19^, glutamate entry into the cells through EAAT3 is functionally related to NCX1 activity^10, 11^. Therefore, once established that NCX1 was essential to mitigate cell injury, we explored whether EAAT3 activity could also be mandatorily required. We initially used the non-transportable EAAT blocker dl-threo-β-benzyloxyaspartic acid (DL-TBOA)^22^ at the concentration of 300 µM^19^. It has been shown that DL-TBOA inhibits ischemia-evoked glutamate release, which has been ascribed to EAATs reverse mode of action^23^. Since this effect could play a protective role during ischemia^24^, we administered DL-TBOA at the onset of the reoxygenation phase (Fig. 1). Under these experimental conditions glutamate failed to attenuate H/R-induced cell damage (Fig. 3a). DL-TBOA per se did not affect cell viability (Figs. 1 and 3a, b). To specifically assess EAAT3 contribution, we used an RNAi-mediated approach (Supplementary Fig. 1). As shown in Fig. 3c, EAAT3 silencing significantly prevented the effect of glutamate, supporting that EAAT3 is determinant in this response.

### Glutamate supplementation and ATP production: role of NCX and EAAT

**Fig. 4:**
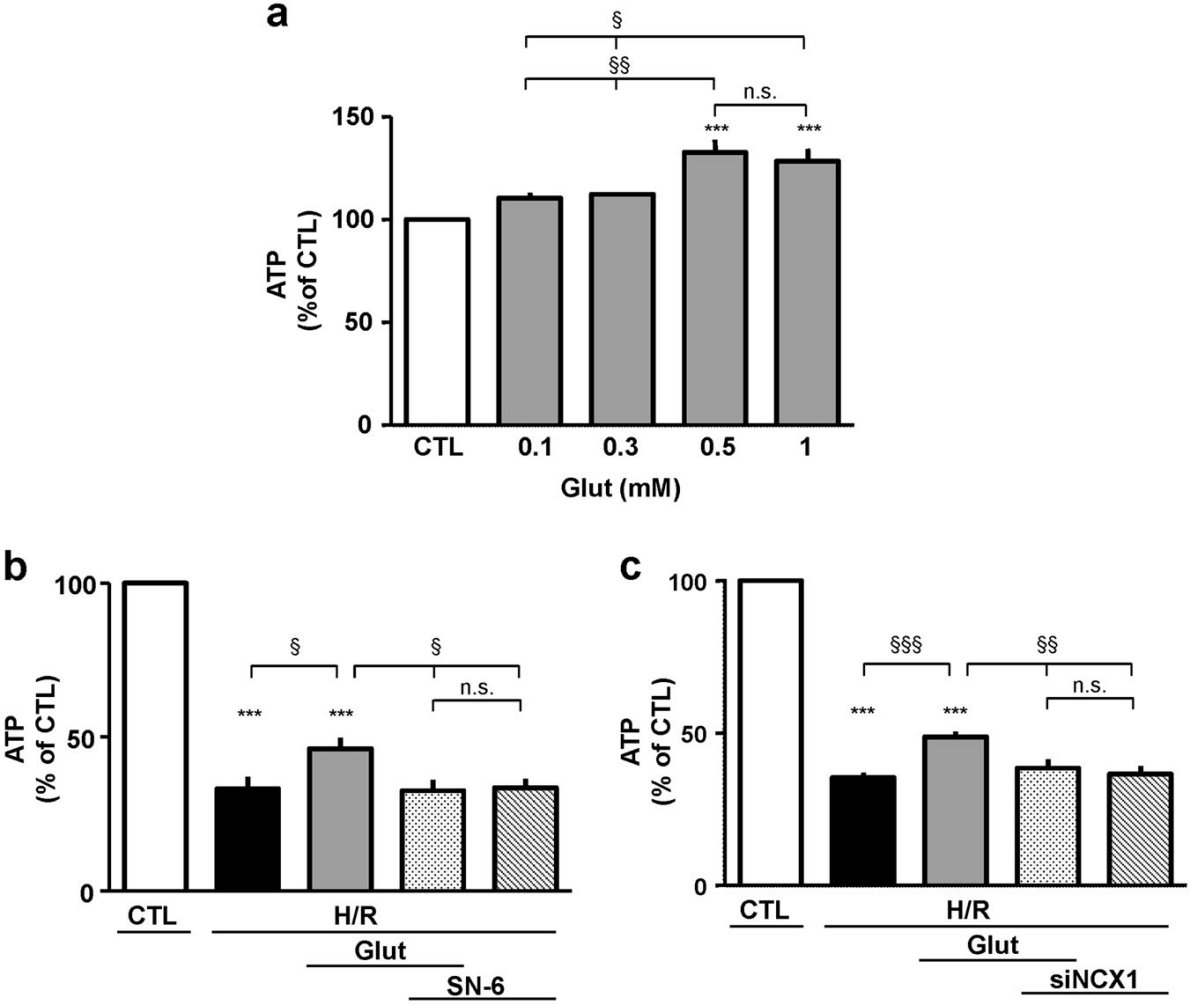
Glutamate-induced recovery of ATP synthesis during H/R challenge is prevented by NCX1 inhibition. **a** Intracellular ATP levels evaluated under normoxic conditions in cells exposed to glutamate (0.1 and 1 mM) for 1 h. ATP levels were normalized to the respective sample protein content and expressed as percentage of the control. Each column represents the mean ± S.E.M. of six independent experiments performed in triplicate. Differences among means were assessed by one-way ANOVA followed by Dunnet’s post hoc test. *F*(4, 25) = 11.38. ***p < 0.0001 vs control group; ^§§^*p* < 0.001, ^§^*p* < 0.01 vs the indicated groups. Both 0.1 and 0.3 mM glutamate concentrations were not significantly different from the control group. **b** Intracellular ATP levels evaluated under different experimental conditions. Glutamate (0.5 mM) was added at the onset of the reoxygenation phase, alone or in combination with 1 μM SN-6, according to the time schedule showed in Fig. 1. ATP was monitored after 1 h. ATP levels were normalized to the respective sample protein content and expressed as percentage of the control. Each column represents the mean ± S.E.M. of almost five independent experiments performed in triplicate. Differences among means were assessed by one-way ANOVA followed by Dunnet’s post hoc test. *F*(5, 48) = 97.62. ***p < 0.0001 vs control group; ^§^*p* < 0.01 vs the indicated groups. The H/R group was not significantly different from the H/R + SN-6 + Glut and H/R + SN-6 groups. **c** Silencing of NCX1 was performed 30 h before H/R challenge. Glutamate was added for the first hour of the reoxygenation phase. ATP levels were normalized to the respective sample protein content and expressed as percentage of the control. Each column represents the mean ± S.E.M. of almost four independent experiments performed in triplicate. Differences among means were assessed by one-way ANOVA followed by Dunnet’s post hoc test. *F*(5, 28) = 189.5. ***p < 0.0001 vs control group; ^§§§^*p* < 0.0001, ^§§^*p* < 0.001 vs the indicated groups. The H/R group was not significantly different from the H/R + siNCX1 + Glut and H/R + siNCX1 groups. H/R hypoxia/reoxygenation, Glut glutamate, siNCX1 siRNA for NCX1, n.s. not significant

**Fig. 5:**
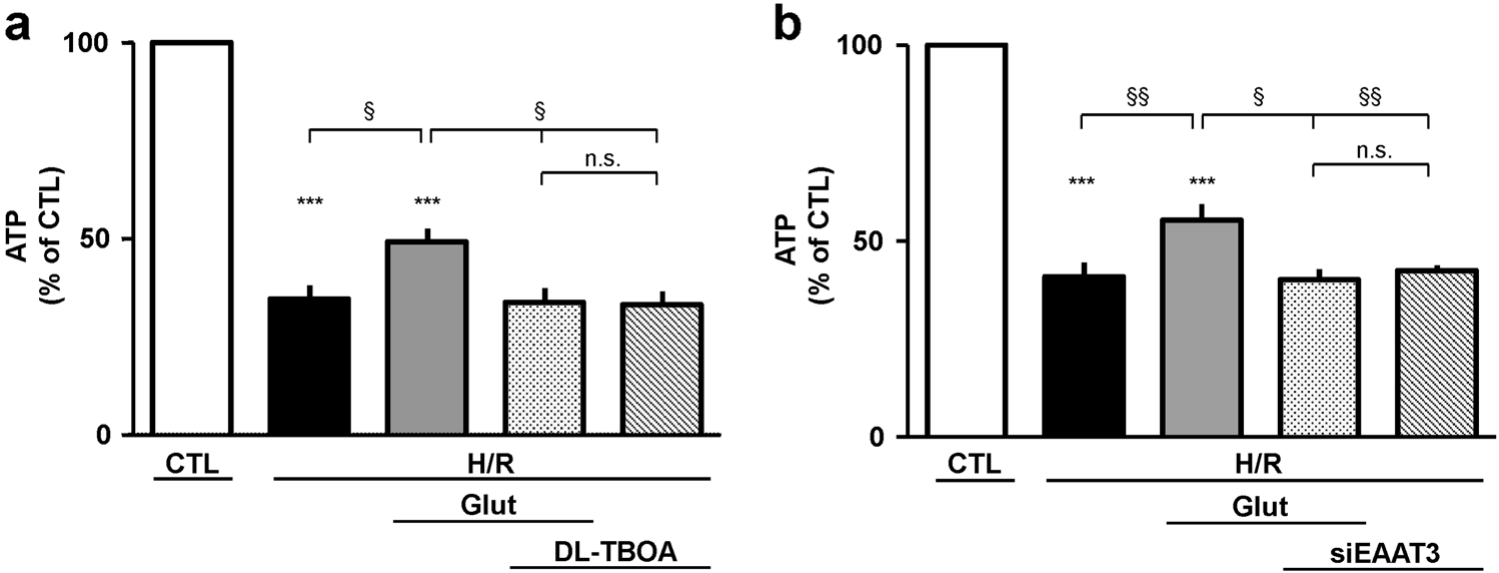
Glutamate-induced recovery of ATP synthesis during H/R challenge is prevented by EAAT3 inhibition. Intracellular ATP levels evaluated under different experimental conditions. **a**  Glutamate (0.5 mM) was added at the onset of the reoxygenation phase, alone or in combination with 300 μM DL-TBOA, according to the time schedule showed in Fig. 1. ATP was monitored after 1 h. ATP levels were normalized to the respective sample protein content and expressed as percentage of the control. Each column represents the mean ± S.E.M. of almost five independent experiments performed in triplicate. Differences among means were assessed by one-way ANOVA followed by Dunnet’s post hoc test. *F*(5, 23) = 65.12. ***p < 0.0001 vs control group; ^§^*p* < 0.01 vs the indicated groups. The H/R group was not significantly different from the H/R + DL-TBOA + Glut and H/R + DL-TBOA groups. **b** Silencing of EAAT3 was performed 30 h before H/R challenge. Glutamate was added during the first hour of the reoxygenation phase. ATP levels were normalized to the respective sample protein content and expressed as percentage of the control. Each column represents the mean ± S.E.M. of almost four independent experiments performed in triplicate. Differences among means were assessed by one-way ANOVA followed by Dunnet’s post hoc test. *F*(5, 18) = 84.31. ***p < 0.0001 vs control group; ^§§^*p* < 0.001, ^§^*p* < 0.01 vs the indicated groups. The H/R group was not significantly different from the H/R + siEAAT3 + Glut and H/R + siEAAT3 groups. H/R hypoxia/reoxygenation, Glut glutamate, siEAAT3 siRNA for EAAT3, n.s. not significant

Under both physiological and pathological conditions glutamate can contribute to cellular energy balance as substrate for anaplerotic reactions, and we demonstrated that NCX1 provides functional support for both glutamate uptake and ATP synthesis^10, 11, 19^. Therefore, we investigated whether the protective effect of glutamate could be ascribed to its potential to sustain ATP synthesis. Firstly, cells were exposed to different concentrations of glutamate (0.1–1 mM) under normoxia for 1 h. We found that glutamate concentrations of 0.5 and 1 mM produced a significant rise in intracellular ATP content (Fig. 4a), in line with our previous findings obtained in undifferentiated SH-SY5Y cells^10^. Thus, for the subsequent sets of experiments we chose the lowest concentration able to significantly increase intracellular ATP levels (0.5 mM). We explored glutamate ability to sustain ATP synthesis during the reoxygenation phase, and verified NCX1/EAAT3 functional involvement. As expected, during H/R challenge intracellular ATP levels sharply dropped (Fig. 4b). When glutamate was given during the first hour of the reoxygenation phase, it evoked a partial recovery of intracellular ATP content. Interestingly, SN-6 counteracted this effect and this finding was confirmed by NCX1 silencing, suggesting that the exchanger played a crucial role during this metabolic response (Fig. 4c). Considering that NCX1/EAAT3 interaction was necessary to confer the partial protection from H/R-induced cell damage, we then explored EAAT3 role. Both DL-TBOA and EAAT3 silencing prevented glutamate-induced ATP recovery (Fig. 5). Both SN-6 and DL-TBOA did not affect ATP levels neither under normoxia^10^ (data not shown) nor after H/R protocol (Figs. 4 and 5).

### Glutamate effect on cellular bioenergetics following H/R challenge

**Fig. 6:**
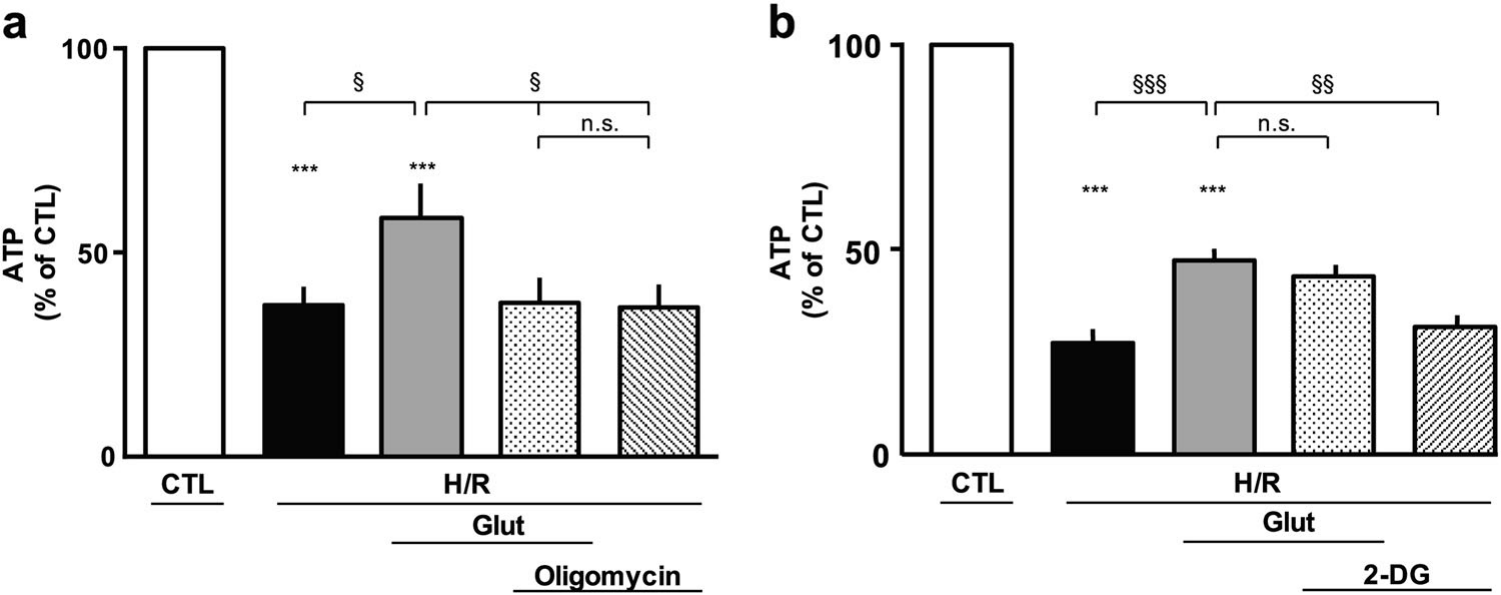
Effect of oligomycin and 2-DG on glutamate-induced ATP synthesis in cells subjected to H/R challenge. **a** Intracellular ATP content evaluated after H/R in the presence of 0.5 mM glutamate, alone or in combination with 3 µg/ml olygomicin, added at the beginning of the reoxygenation phase and maintained for 1 h. ATP levels were normalized to the respective sample protein content and expressed as percentage of the control. Each column represents the mean ± S.E.M. of five independent experiments performed in triplicate. Differences among means were assessed by one-way ANOVA followed by Dunnet’s post hoc test. *F*(5, 42) = 27.40. ***p < 0.0001 vs control group; ^§^*p* < 0.01 vs the indicated groups. The H/R group was not significantly different from the H/R + oligomycin + Glut and H/R + oligomycin groups. **b** Intracellular ATP content evaluated after H/R in the presence of 0.5 mM glutamate, alone or in combination with 2 mM 2-DG, added at the beginning of the reoxygenation phase and maintained for 1 h. ATP levels were normalized to the respective sample protein content and expressed as percentage of the control. Each column represents the mean ± S.E.M. of seven independent experiments performed in triplicate. Differences among means were assessed by one-way ANOVA followed by Dunnet’s post hoc test. *F*(5, 35) = 99.91. ***p < 0.0001 vs control group; ^§§§^*p* < 0.0001, ^§§^*p* < 0.001 vs the indicated groups. The H/R group was not significantly different from the H/R + 2-DG group. H/R hypoxia/reoxygenation, Glut glutamate, 2-DG 2-deoxyglucose, n.s. not significant

An increase in ATP levels may occur within the cells by two main bioenergetics pathways: the oxidative phosphorylation and the glycolysis. Therefore, we tested whether the protection induced by glutamate during H/R was related to an improvement of the oxidative metabolism or to an enhancement of the glycolytic pathway. We performed a set of experiments using the ATP synthase inhibitor oligomycin (3 µg/ml) or the glycolysis inhibitor 2-deoxyglucose (2-DG, 2 mM)^25^. We found that oligomycin counteracted the increase in ATP levels induced by glutamate (Fig. 6a), whereas 2-DG was unable to prevent this effect (Fig. 6b). These data supported the hypothesis that in our experimental setting, the mitochondrial oxidative phosphorylation may be the route to produce ATP in the presence of glutamate. Both oligomycin and 2-DG did not affect ATP levels neither under normoxia (data not shown) nor after H/R protocol (Fig. 6).

### Analysis of NCX and EAAT expression following H/R challenge

**Fig. 7:**
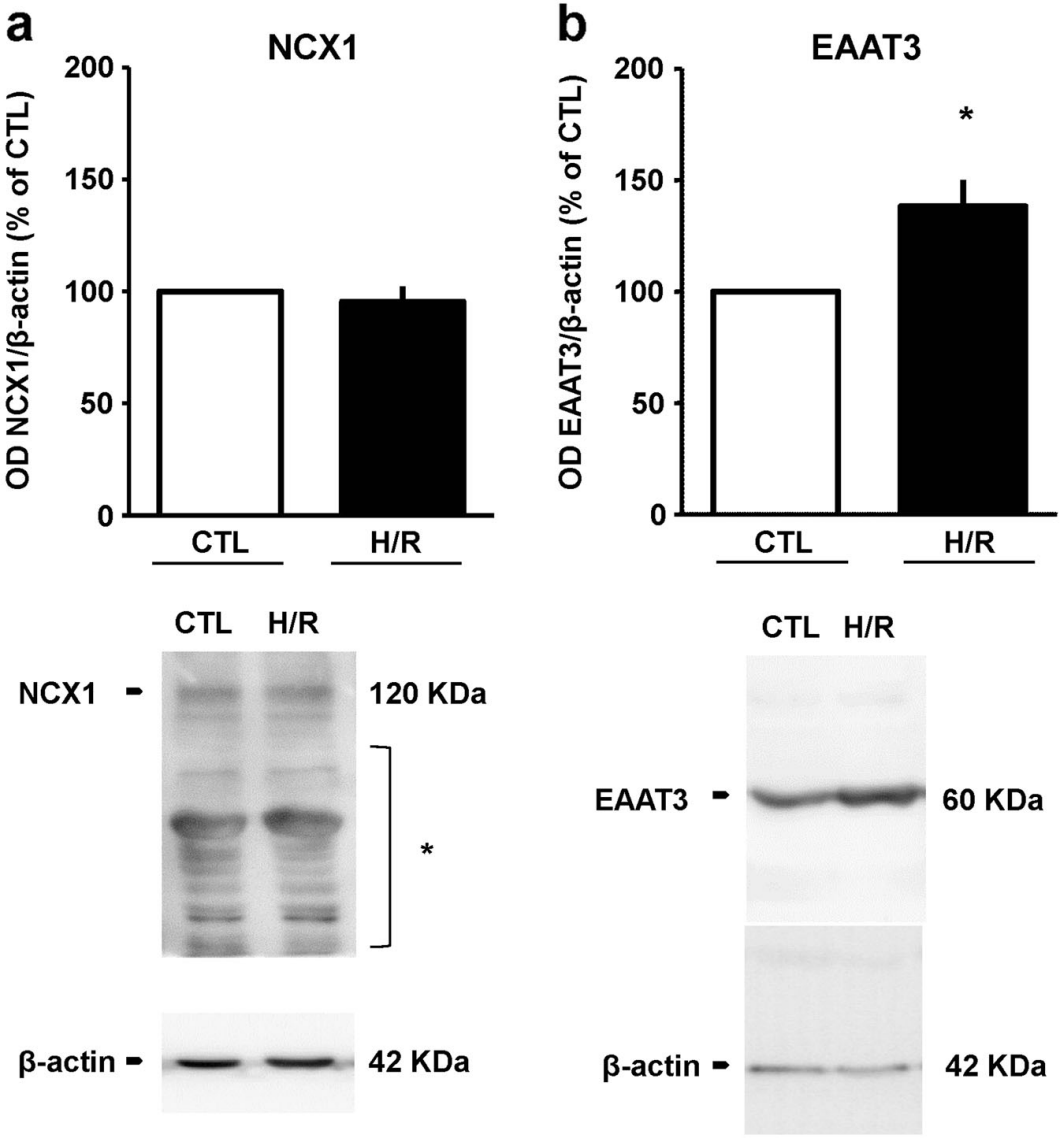
NCX1 and EAAT3 expression after H/R challenge Quantitative densitometry showing (**a**) the expression of NCX1 and (**b)** the Na^+^-dependent glutamate transporters EAAT3 in cells exposed to H/R. β-actin was used as loading control. Normalized optical density values are expressed as percentage of the respective control. Each column represents the mean ± S.E.M. of three independent experiments. Differences among means were assessed by Student’s *t*-test. **b** *p < 0.05 vs CTL. Representative western blot images are shown below. CTL control, H/R hypoxia/reoxygenation; asterisk indicates cross-reactive bands

Once verified that under H/R conditions NCX1 and EAAT3 were critical to support the protective action of glutamate, we investigated whether the expression of these proteins was modified by H/R challenge. Interestingly, our results showed that NCX1 expression was unmodified, whereas EAAT3 levels were significantly increased (approximately 40% compared to control group; Fig. 7a, b).

### Analysis of NCX activity alteration following H/R injury

**Fig. 8:**
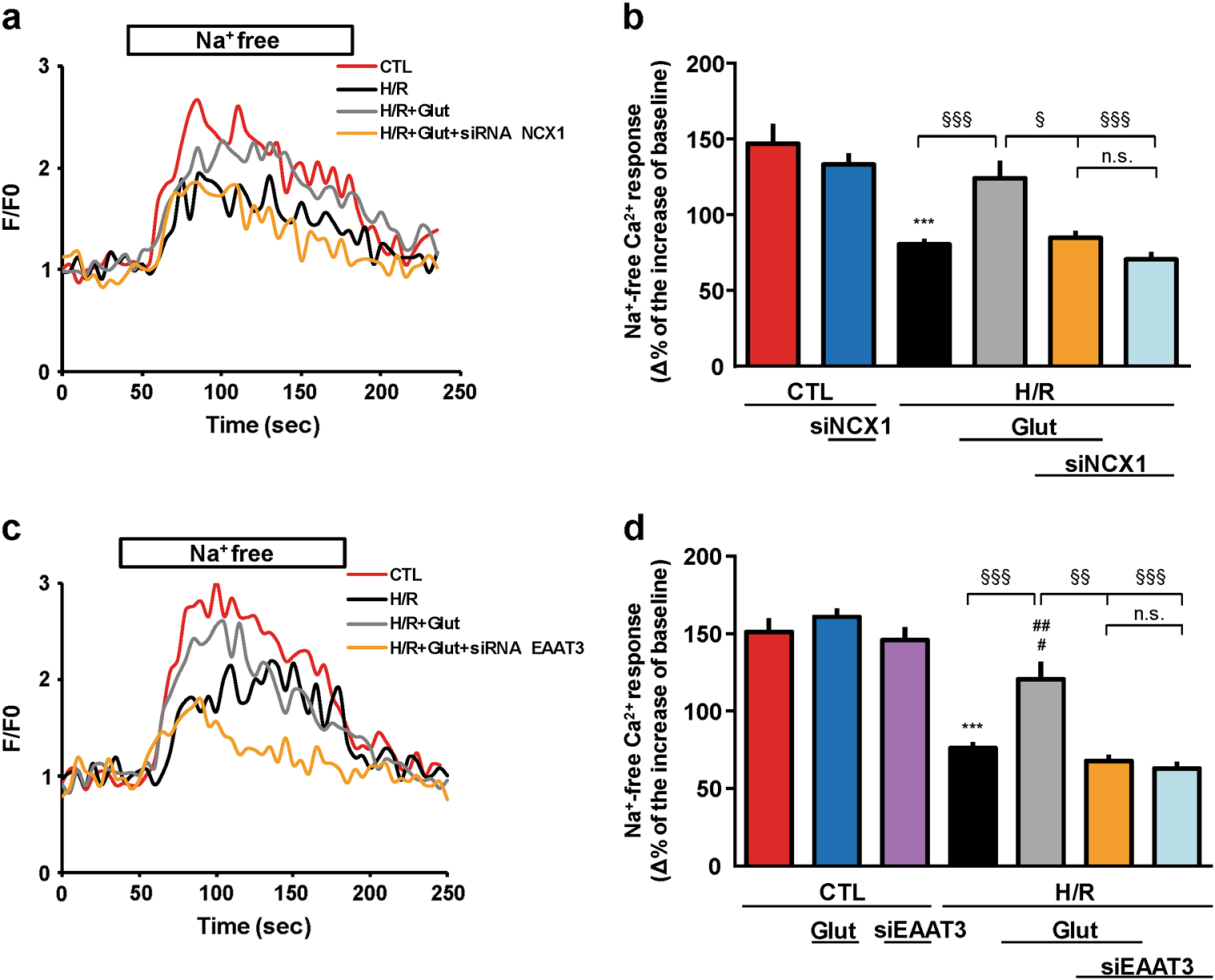
Effect of glutamate exposure on NCX activity after H/R challenge. **a**, **c** Single trace recordings of Ca^2+^ response to Na^+^ free after incubation under normoxic conditions (red line), H/R insult (black line), and H/R protocol with Glut supplementation (0.5 mM) during the reoxygenation phase alone (gray line) or in combination with siRNA (siNCX1 and siEAAT3, orange line). Fluorescence intensity values were normalized to resting fluorescence (*F*/*F*0). **b**, **d** Measurements were performed at the end of the experimental protocol as described in Fig. 1. NCX activity was expressed as Δ% fluorescence increase. The bar plot reports the mean ± S.E.M. of fluorescence increase elicited by Na^+^ free pulse. For each experimental group, Δ% values used for the statistical analysis derived from four independent experiments and 100–200 cells were recorded in each different session. Differences among means were assessed by one-way ANOVA followed by Dunnet’s post hoc test. **b** *F*(5, 599) = 18.09. ***p < 0.0001 vs control groups; ^§§§^*p* < 0.0001, ^§^*p* < 0.01 vs the indicated groups. The H/R group was not significantly different from the H/R + siNCX1 + Glut and H/R + siNCX1 groups. The H/R + Glut group was not significantly different from control groups. **d** *F*(6, 830) = 32.10. ***p < 0.0001 vs control groups; ^##^*p* < 0.001 vs CTL + Glut; ^#^*p* < 0.01 vs CTL; ^§§§^*p* < 0.0001, ^§§^*p* < 0.001 vs the indicated groups. The H/R group was not significantly different from the H/R + siEAAT3 + Glut and H/R + siEAAT3 groups. The H/R + Glut group was not significantly different from CTL + siEAAT3. CTL control, H/R hypoxia/reoxygenation, Glut glutamate, siNCX1 siRNA for NCX1, siEAAT3 siRNA for EAAT3, n.s. not significant

Under ischemic conditions NCX activity can be altered as a consequence of energy metabolism failure^26^; therefore, we investigated whether NCX activity could be affected by our experimental conditions (Fig. 1). Exchanger activity was evaluated as Na^+^ gradient-dependent Ca^2+^ uptake in Fluo-4-loaded cells, by monitoring fluorescence signals. We analyzed reverse-mode activity triggered by a stepwise reduction of extracellular Na^+^ (substituted with K^+^) at the end of the experimental protocol. When NCX reverse mode was activated, a decrease in fluorescence signal (approximately 50%) occurred in H/R cells compared to the controls (Fig. 8). Notably, glutamate exposure elicited a significant increase in fluorescence signal compared to H/R group. This effect was lost when cells were pre-treated with siNCX1 or siEAAT3.

siRNA treatment did not affect per se Ca^2+^ responses (Fig. 8b, d). Likewise, fluorescence levels were not altered by glutamate supplementation under normoxia.

## Discussion

By using RA-differentiated SH-SY5Y neuroblastoma cells, we showed for the first time that glutamate—given at the onset of the reoxygenation phase—can improve cell viability in an in vitro model of H/R. We determined that the protective action of glutamate likely depends on its ability to stimulate the resumption of oxidative metabolism. Importantly, glutamate effect strictly relies on NCX1 activity.

It has been known for several years that during ischemic brain injury, excessive release of glutamate occurs, culminating in neuron destruction as a consequence of the overstimulation of glutamate receptors, especially NMDA receptors (NMDARs)^27^. However, all the clinical trials of NMDAR antagonists for stroke and traumatic brain injury surprisingly showed a lack of efficacy of these compounds^28^. These results suggest that glutamate may be involved in the early neurodestructive phase after ischemic injury^28^, but when the physiological conditions are restored, glutamate may cease its detrimental role and participate in cell recovery through its known physiological functions. Glutamate is a key compound in cellular metabolism, as it can fuel respiration and improve the anaplerotic refilling of TCA cycle intermediates^6^. We previously found that under normoxia glutamate can increase ATP levels in neuronal and glial cells^10, 11^. Interestingly, we recently demonstrated that in cardiomyocytes, glutamate can sustain cell metabolism also in a setting of H/R^19^. In both cases glutamate effect requires NCX activity.

In neuronal models of I/R, the role of the different NCX isoforms has been widely studied, however it still remains controversial and incompletely elucidated. On the one hand, Pignataro et al^29^. showed that in a model of permanent focal cerebral ischemia, the reduction in NCX1 and NCX3 protein expression causes an increase in the ischemic infarct, suggesting the importance of the NCX forward mode of operation in extruding Ca^2+^ in the ischemic brain^29^. On the other hand, SEA0400, a selective inhibitor of reverse-mode NCX1, was given at reperfusion and significantly reduced the infarct volume in the cortex after 24 h of reperfusion that followed 2 h of ischemia^30^. These findings suggest that NCX activity depends on the specific pathological context within which the exchanger works, therefore, tight regulation of its activity is critical for the brain. Under our experimental conditions, we did not observe a significant role of NCX during the entire H/R protocol. However, we interestingly found that NCX became crucial for the functional support of glutamate-induced protection. In particular, when cells were exposed to H/R challenge the viability was dramatically affected. Exposure to glutamate during the entire reoxygenation phase induced a partial but significant cell survival recovery, which was abolished by the NCX inhibitor SN-6. By using an RNAi approach, we showed that NCX1 was functionally involved in such glutamate-induced protection.

H/R causes a dramatic fall in the intracellular ATP content, as a consequence of the energy imbalance^31^. Therefore, a question arose as to whether glutamate could fuel oxidative metabolism and stimulate ATP synthesis, thereby improving cell viability. During our H/R protocol, ATP levels dramatically dropped. Exposure to glutamate for the first hour of the reoxygenation caused a significant rise (50%) in the intracellular ATP content, compared to H/R, suggesting that the positive effect exerted by glutamate may rely on its ability to support cell metabolism. Glutamate also serves as the precursor for the synthesis of GABA, and its uptake through EAAT3 may be relevant to this purpose^32, 33^. Since SH-SY5Y cells can synthesize and release this inhibitory neurotransmitter^34^, we cannot rule out that, at least in part, the observed neuroprotective effect of glutamate may rely on its conversion to GABA. Its production by SH-SY5Y is far less than that observed in brain areas enriched in GABAergic neurons^34^, therefore, our model—not entirely reflecting in vivo central nervous system complexity—does not allow us to further clarify this issue.

To find the source responsible for glutamate-induced ATP generation, we investigated both oxidative phosphorylation and glycolysis processes. Exposure to oligomycin counteracted the glutamate-induced increase in ATP levels, whereas the presence of 2-DG was ineffective in blocking ATP synthesis. These results suggested the possibility that the increase in intracellular ATP content induced by exogenous glutamate might depend on its ability to fuel the mitochondrial oxidative phosphorylation. Our hypothesis of glutamate as an alternative energy source in metabolism-compromised states is in line with recent findings that try to metabolically “rehabilitate” the excess of glutamate released during the early phase of ischemia. It has been shown that the correction of hypoxia by supplemental oxygen during cerebral ischemia can induce the expression of the glutamate-metabolizing enzyme “glutamate oxaloacetate transaminase” (GOT)^35^, which in turn can utilize the otherwise neurotoxic glutamate and maintain cellular energetics in a setting of hypoglycemia^6^. As a transaminase, GOT catalyzes the transfer of the amino group from glutamate to oxaloacetate, to generate aspartate and alpha-ketoglutarate, which fuels the TCA cycle and sustains cell viability. The increased GOT activity has been indirectly demonstrated through the rise in intracellular aspartate levels. This point could be a matter of concern, raising the question of a possible aspartate neurotoxicity through the NMDAR activation. However, the fact that GOT overexpression reduced ischemic stroke lesion volume, attenuated neurodegeneration, and improved post stroke sensorimotor function^6^, lead us to hypothesize that the excess of aspartate is mainly retained in the cytosol, where it is metabolically recycled^36^ without causing significant toxicity. The modulation of the glutamate dehydrogenase (GDH) enzyme has also been proposed. GDH is a TCA cycle enzyme that converts glutamate to alpha-ketoglutarate. Under conditions of high energy demand (i.e., I/R and oxygen-glucose deprivation, OGD), the modulation of GDH activity toward the utilization of glutamate seems to increase ATP production and to positively affect neuronal viability^5^. One possible explanation is that the activation of GDH may increase the influx of alpha-ketoglutarate in the TCA cycle, thereby reducing the extracellular glutamate release. Collectively, our approach fits into this novel scenario, which reveals the potentiality of glutamate to be transformed into a neuronal “survival factor”.

We know from our previous studies that glutamate can obtain access to the mitochondrial matrix through EAAT3/EAAC1, whose activity is supported by reverse-mode NCX1^10, 11, 19^. Here we report that the inhibition of EAATs with DL-TBOA blocked the beneficial effects of glutamate, likely by hampering glutamate entry. By using a specific RNAi approach, we demonstrated that EAAT3 is the isoform specifically involved in this pathway. As aforementioned, the main interest in EAAT3/EAAC1 takes origin from our previous study^11^, in which immunoprecipitation experiments performed on rat brain extracts demonstrated a specific link between NCX1 and EAAC1, without any involvement of the glial glutamate transporters GLT-1 and GLAST^11^. Glutamate turnover is actually dependent on glial compartment^33, 37^ and, in this context, GLT-1 is the main transporter modulating extracellular glutamate levels^33, 38, 39^. Considering that our model only recapitulates neuronal functions, we cannot exclude that in in vivo systems GLT-1 may contribute to the metabolic response evoked by glutamate in ischemic settings. Further research is needed to shed more light on this point.

Overall, the data presented here indicated that glutamate may attenuate neuronal damage in an in vitro setting of H/R and that NCX1 and EAAT3 have a central role. In relation to these findings, we also analyzed the expression of both NCX1 and EAAT3. Remarkably, we found an increase in EAAT3 expression, in line with previous reports showing a specific rapid increase in EAAC1 expression after transient focal ischemia in rat pyramidal neurons of the hippocampal CA1 region and of the parietal cerebral cortex^40^. This result may reflect the peculiar role of EAAT3/EAAC1 in subserving a neuroprotective mechanism in which the uptake of glutamate and its fate within cells play a major role. In this respect, EAAC1 knockout has been reported to reduce the brain tolerance in a murine model of focal brain ischemia^41^. Although the authors ascribe such vulnerability to impaired cysteine uptake by EAAC1^42^—with a consequent lack in antioxidant defenses—they fail to identify any significant change in glutathione levels between knockout and wild-type mice. This finding suggests that the absence of EAAT3/EAAC1 may likely worsen the focal brain ischemia outcomes because of the impaired glutamate entry, which may negatively affect neuronal metabolism. On the other hand, we cannot overlook the peculiar activity of EAAT3/EAAC1 as cysteine transporter, since other results supported its role in maintaining the redox state of neurons^43–45^. In particular, in neurons from EAAC1 knockout animals, pre-treatment with the membrane-permeant prodrug *N*-acetyl cysteine^43–45^ counteracts the increased vulnerability to I/R-induced oxidative stress. Therefore, we cannot exclude such a role for EAAT3/EAAC1 also in our experimental model. Further in vivo studies are needed to better elucidate the balance among the glutamate/cysteine transport activity of EAAT3 in ischemic settings. As for NCX1, we did not observe any change in its expression, in line with previous reports analyzing the expression of the exchanger in a comparable model of H/R^46^. Nevertheless, we sought to explore the possible impact of the H/R challenge on the reverse-mode activity of the exchanger. Surprisingly, NCX reverse-mode activity was significantly reduced and glutamate supplementation tended to normalize the global exchanger activity.

Regarding the decrease in the exchanger reverse-mode activity, several mechanisms may come into play, including the removal of the exchanger from the extracellular surface by endocytic pathways^47, 48^ and the compensatory action possibly taking place among the different isoforms^49^. Nevertheless, the increase in the intracellular ATP levels induced by glutamate ultimately represents an upstream signal that tends to restore the overall cellular homeostasis. RNAi directed against NCX1 specifically abolished the partial recovery of NCX activity induced by glutamate exposure, and the use of a specific EAAT3 small interference RNA (siRNA) confirmed that the glutamate effects were mediated by the NCX1-EAAT3 interplay.

Taken together, these findings validate that the defensive glutamate action relied on the activity of NCX1 that supported EAAT3 in a mandatory way. As already stated in our previous reports^10, 11, 19^, we propose that by extruding Na^+^, the reverse-mode activity of the exchanger is essential for maintaining the Na^+^-driving force for effective glutamate uptake. This uptake in turn tends to bring Ca^2+^ into mitochondria and to increase ATP synthesis, likely through enhancement of mitochondrial dehydrogenases activity^50^. Accordingly, the reduction in the exchanger reverse-mode activity could strongly affect the ability of EAAT3 to efficiently uptake glutamate. This effect in turn may reduce the amount of glutamate that is metabolically available and may explain why we did not observe a full recovery of both ATP production and cell viability. However, other mechanisms may also explain the partial efficacy of glutamate, including a slight excitotoxic effect mediated by NMDAR^51, 52^ and/or the activation of an NMDAR-independent oxidative response^53^. To confirm these hypotheses, further investigations are needed.

In conclusion, in this report, we have demonstrated that glutamate given at the onset of the reoxygenation phase can improve cell viability in an in vitro neuronal model of H/R. The present findings suggest that the ability of glutamate to possibly serve as a “survival factor” relies, at least in part, on its metabolic utilization that fuels the TCA cycle and the oxidative phosphorylation, leading to an increase in ATP content. Importantly, glutamate action depends on the coordinated activity of NCX1 and EAAT3, which, in a complementary fashion, orchestrate its uptake and intracellular utilization. We have in mind that it may be difficult to translate our in vitro results in in vivo models. However, the information presented here could be helpful in the refinement of further in vivo studies, which could pave the way for a change in the classical view of glutamate as detrimental factor.

## Materials and methods

### Cell culture

Human neuroblastoma cell line SH-SY5Y^54^ was obtained from American Type Culture Collection (CRL-2266). SH-SY5Y cells were cultured in 75-ml vented culture flasks using Eagle’s minimum essential medium/nutrient mixture Ham’s F-12 (1:1) media supplemented with 10% fetal bovine serum (FBS), 100 U/ml penicillin, and 100 μg/ml streptomycin. The cell culture medium was replaced every 2 days. The cells were maintained in a humidified incubator at 37 °C and 5% CO_2_.

Differentiation into neuron-like cells was achieved by treatment with 10 µM all-*trans* RA^55, 56^ that was added to the cell culture medium every 3 days for 1 week prior to perform the experiments.

For lactate dehydrogenase (LDH) and NCX activity assays, differentiated cells (4 × 10^5^ cells/well) were cultured onto 6-well plates, whereas for ATP assay differentiated cells (2.5 × 10^4^ cells/well) were cultured onto 96-well plates.

### Silencing of NCX1, NCX3, and EAAT3

RNAi was performed as previously described^57^ with minor modifications. Specifically, silencing of NCX1, NCX3, and EAAT3 was performed with HyPerfect Transfections Kit (Qiagen) according to the manufacturer’s instruction by using FlexiTube siRNA for NCX1 (Qiagen, Hs_SLC8A1_9), FlexiTube siRNA for NCX3 (Qiagen Hs_SLC8A3_7), and FlexiTube siRNA for EAAT3 (Qiagen Hs_SLC1A1_3). The validated irrelevant Allstars siRNA (Qiagen) was used as a negative control. Target sequence of the FlexiTube NCX1 siRNA was Hs_SLC8A1_9 (5′-CAGGCCATCTTCTAAGACTGA-3′), of the NCX3 siRNA sequences was Hs_SLC8A3_7 (5′-ACCATTGGTCTCAAAGATTCA-3′), and of the EAAT3 siRNA was Hs_SLC1A1_3 (5′-GAGGACTGTTCTAACTAGTAA-3′). The transfection protocol was as follows: SH-SY5Y cells (90.000 cells/well) were differentiated with 10 µM RA in 12-well plates. After 7 days, SH-SY5Y were incubated 48 h with 2.3 ml of MEM/F-12 media containing 100 µl of MEM/F-12 (without FBS and antibiotics), 12 µl of HyPerfect Transfection Reagents, and 80 nM of each siRNA oligonucleotide (each well). Thirty hours after transfection, SH-SY5Y were subjected to H/R protocol. Cells were then tested for viability, ATP content, NCX activity, or protein expression. The yield of RNA silencing was assessed by western blot analysis by using specific antibodies.

### In vitro H/R challenge

The cells were subjected to H/R as described earlier^58^ with minor modifications. Briefly, an OGD buffer containing (in mM): 154 NaCl; 5.6 KCl; 5.0 HEPES; 3.6 NaHCO_3_; and 2.3 CaCl_2_ (pH 7.4) was bubbled with 95% N_2_–5% CO_2_ for 10 min at room temperature. Then, the culture medium containing 4.5 g/l glucose was replaced with the OGD buffer and the culture plates were put into an airtight chamber gassed with 95% N_2_–5% CO_2_ for 10 min. The chamber was then sealed and transferred to a humified incubator at 37 °C, 5% CO_2_, for 16 h. Reoxygenation was initiated by opening the chamber and returning the cells to their normal culture conditions for 24 h before they were used to perform experiments. Preliminary time-course studies showed that a 16 h period of hypoxia followed by 24 h of reoxygenation was optimal to achieve a significant increase in cell death (data not shown).

### Evaluation of cell viability

Cell injury was evaluated by the measurement of LDH activity released from the cytosol of damaged cells in the experimental media^59, 60^ and by the method of double staining with fluorescein diacetate/propidium iodide (FDA/PI)^25, 60^. Briefly, culture medium was collected at the end of the H/R challenge and centrifuged at 250 × *g* for 10 min. Then, 100 µl of the supernatant were transferred to a 96-well plate and incubated with the same volume of reaction mixture (Diaphorase/NAD^+^ mixture premixed with iodotetrazolium chloride/sodium lactate (Roche Diagnostics, Monza, Italy) at room temperature in dark environment for 30 min.

LDH activity was assessed by reading the absorbance of the sample medium at 490 nm in a Victor Multilabel Counter plate reader (Perkin Elmer, Waltham, MA, USA). For FDA/PI staining, cells were differentiated on glass coverslips and subjected to H/R. Afterwards, cells were treated with 36 μM FDA (Sigma, Milan, Italy) and 7 µM PI (Calbiochem., San Diego, CA, USA) for 10 min at room temperature in phosphate-buffered saline (PBS). Stained cells were examined immediately with an inverted Zeiss Axiovert 200 microscope (Carl Zeiss, Milan, Italy) and then analyzed. When FDA crosses the cell membrane it is hydrolyzed by intracellular esterases producing a green-yellow fluorescence. Cell damage curtails FDA staining and allows cell permeation by PI that, interacting with nuclear DNA, yields a bright red fluorescence.

### ATP assay

ATP synthesis was evaluated by using a commercially available luciferase-luciferin system (ATPlite, Perkin Elmer, Waltham, MA) according to the manufacturer’s instructions^19^. Cells were differentiated in 96-multiwell plates at the density of 2.5 × 10^4^ cells/well. After 7 days, differentiation medium was removed and the cells were first washed twice with PBS, then exposed to different glutamate concentrations (0.1–1 mM) in Eagle’s minimum essential medium/nutrient mixture Ham’s F-12 (1:1) without FBS, for 1 h at 37 °C. For H/R experiments, after being differentiated and subjected to 16 h of hypoxia in OGD buffer, the reoxygenation phase was started by returning the cells to their normal culture conditions. Glutamate and the specific pharmacological/molecular tools were added as described in the specific time schedule (Fig. 1). After 1 h of reoxygenation, intracellular ATP levels were analyzed with a luminescence counter (Victor Multilabel Counter, Perkin Elmer) and normalized to the respective protein content.

### Antibodies

NCX1 protein was detected by using a commercially available mouse monoclonal IgG antibody^10, 11^ (R3F1, Swant, Bellinzona, Switzerland, dilution 1:500). To dectect EAAC1/EAAT3 protein we used  mouse anti-EAAC1/EAAT3 (Chemicon International, CA, USA, dilution 1:1000)[10-11]. β-actin^59^ (1:10 000; A5316, Sigma) was used as loading control.

### Western blot analysis

Differentiated SH-SY5Y cells were lysed using a protein lysis buffer containing (in mM): 150 NaCl; 10 Tris-HCl (pH 7.4); 1 EDTA (pH 8.0); 1% SDS; and a protease inhibitor cocktail mixture (Roche Diagnostics). Protein content was determined by the Bradford method^54^ (Bio-Rad, Milan, Italy), using bovine serum albumin as standard. Samples containing equal amounts of protein (30 μg) were boiled in 4× Laemmli sample buffer with 2–mercaptoethanol for 10 min. Proteins were subjected to 8% SDS-polyacrylamide gel electrophoresis^61^ and then electro-transferred to polyvinylidine difluoride membranes (Immobilon Transfer Membranes, Millipore Co., Bedford, MA, USA). The membranes were blocked in PBS buffer containing 5% non-fat dry milk for 1 h at room temperature and then incubated with the appropriate primary antibody overnight at 4 °C. Immunoreactions were revealed by incubation with secondary antibody conjugated to horseradish peroxidase (Santa Cruz, CA, USA) (dilution 1:1000) for 1 h at room temperature. An enhanced chemiluminescence detection system (Super Signal West Femto kit, Thermo Scientific, Milano, Italy) was used to detect bound antibodies. Images were captured and stored on a ChemiDoc station (Bio-Rad, Milan, Italy). NCX1 and EAAT3 band densities were analyzed with the Quantity One (Bio-Rad) analysis software and normalized to corresponding β-actin band densities.

### Analysis of NCX activity

#### Solutions

Ca-PSS contained (in mM): 140 NaCl; 5 KCl; 1 MgCl_2_; 10 glucose; 2 CaCl_2_; and 20 HEPES, buffered to pH 7.4 with Tris. Na-PSS had an identical composition except that calcium was omitted and 0.1 mM EGTA was included. K-PSS contained (in mM): 140 KCl; 1 MgCl_2_; 10 glucose; 0.1 CaCl_2_; and 20 HEPES, buffered to pH 7.4 with Tris.

#### Experimental protocol

Intracellular Ca^2+^ levels were measured by single-cell computer-assisted videoimaging using a LSM 510 confocal system (Carl Zeiss, Milan, Italy)^62^. After being differentiated into neuron-like cells on 25 mm coverslip, SH-SY5Y were subjected to H/R challenge (Fig. 1). Afterwards, cells were loaded with 4 µM Fluo-4/AM (Molecular Probe, Eugene, OR) in Ca-PSS 0.08% pluronic acid (Molecular Probe) for 40 min in the dark at room temperature. Coverslips were then washed once in Na-PSS and treated for 10 min with 1 μM thapsigargin in Na-PSS. After an additional wash, coverslips were placed into a perfusion chamber mounted onto the stage of an inverted Zeiss Axiovert 200 microscope. NCX activity was evaluated as Ca^2+^ uptake through the reverse mode by switching Na-PSS to K-PSS. Bath solutions were changed with a peristaltic pump and images were acquired every 5 s. Cells and perfusion solutions were maintained at 37 °C by using a heated microscope stage and climate box form PeCon GmbH. Excitation light was provided by argon laser at 488 nm and the emission was time-lapsed recorded at 505–530 nm. Analysis of fluorescence intensity was performed off-line after images acquisition, by averaging the fluorescence intensity values within selected areas overlying the cell somata as previously described^59, 63^.

#### Drugs and chemicals

SN-6 and DL-TBOA were obtained from Tocris. All the other chemicals were of analytical grade and were purchased from Sigma.

### Data analysis

Data were expressed as mean ± S.E.M. Values < 0.05 were considered to be significant. When comparing two data sets, the Student’s *t*-test for unpaired data was used. To compare multiple groups, statistical comparisons were performed by one-way analysis of variance followed by Dunnet’s post hoc test. Statistical comparisons were carried out using the GraphPad Prism 5 software (GraphPad Software Inc., San Diego, CA).

## Electronic supplementary material

Supplementary Fig. 1

Supplementary Fig. 2

Supplementary information
