# Supplementary figures and images for: Glutamate as a potential “survival factor” in an in vitro model of neuronal hypoxia/reoxygenation injury: leading role of the Na+/Ca2+ exchanger
Source: Cell Death Dis. 2018 Jun 28;9(7):731. doi: 10.1038/s41419-018-0784-6 (PMC6023866; doi:10.1038/s41419-018-0784-6)

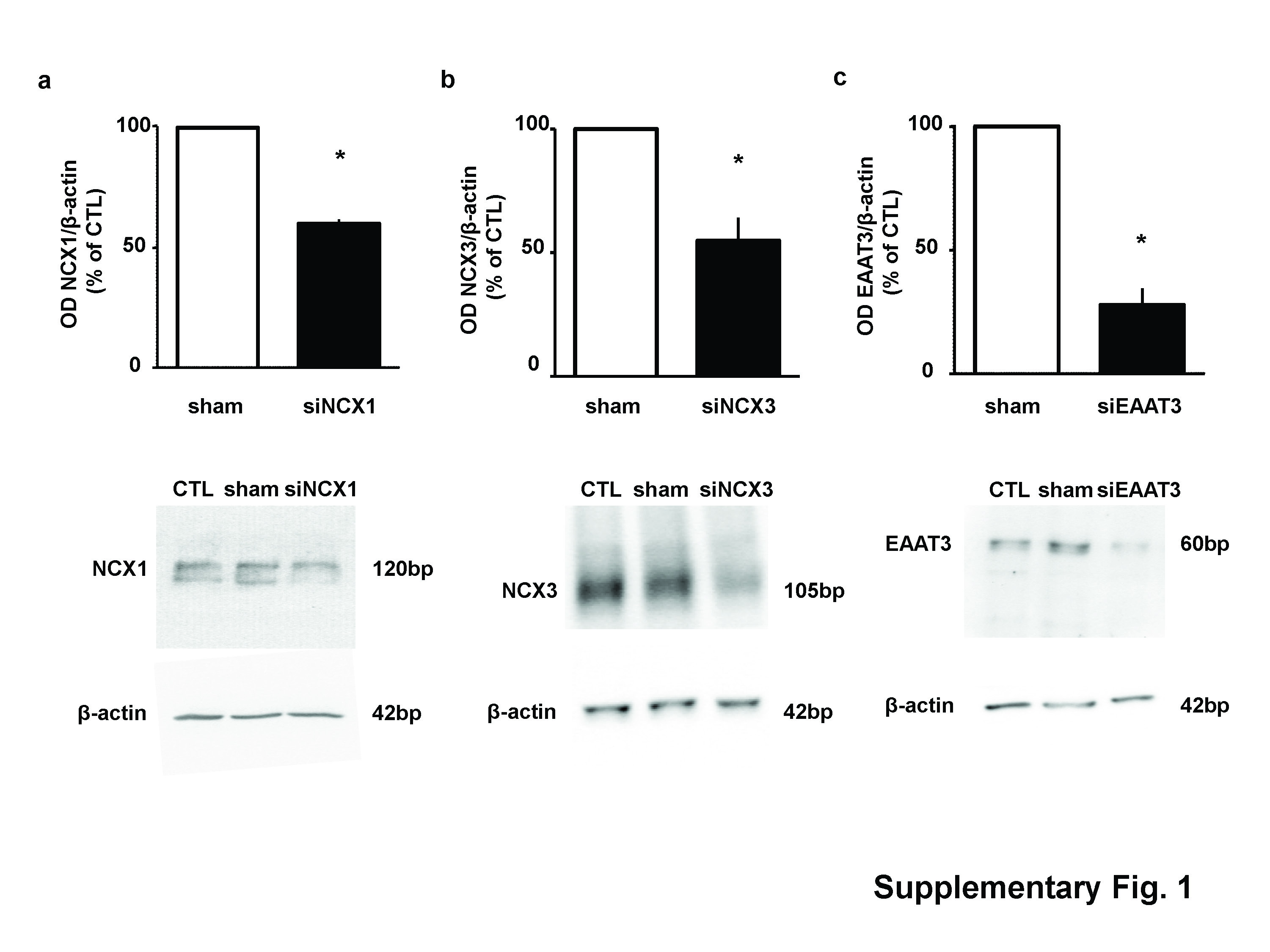

Supplement: Supplementary file 1 — Supplementary Fig. 1 [file 41419_2018_784_MOESM1_ESM.jpg]

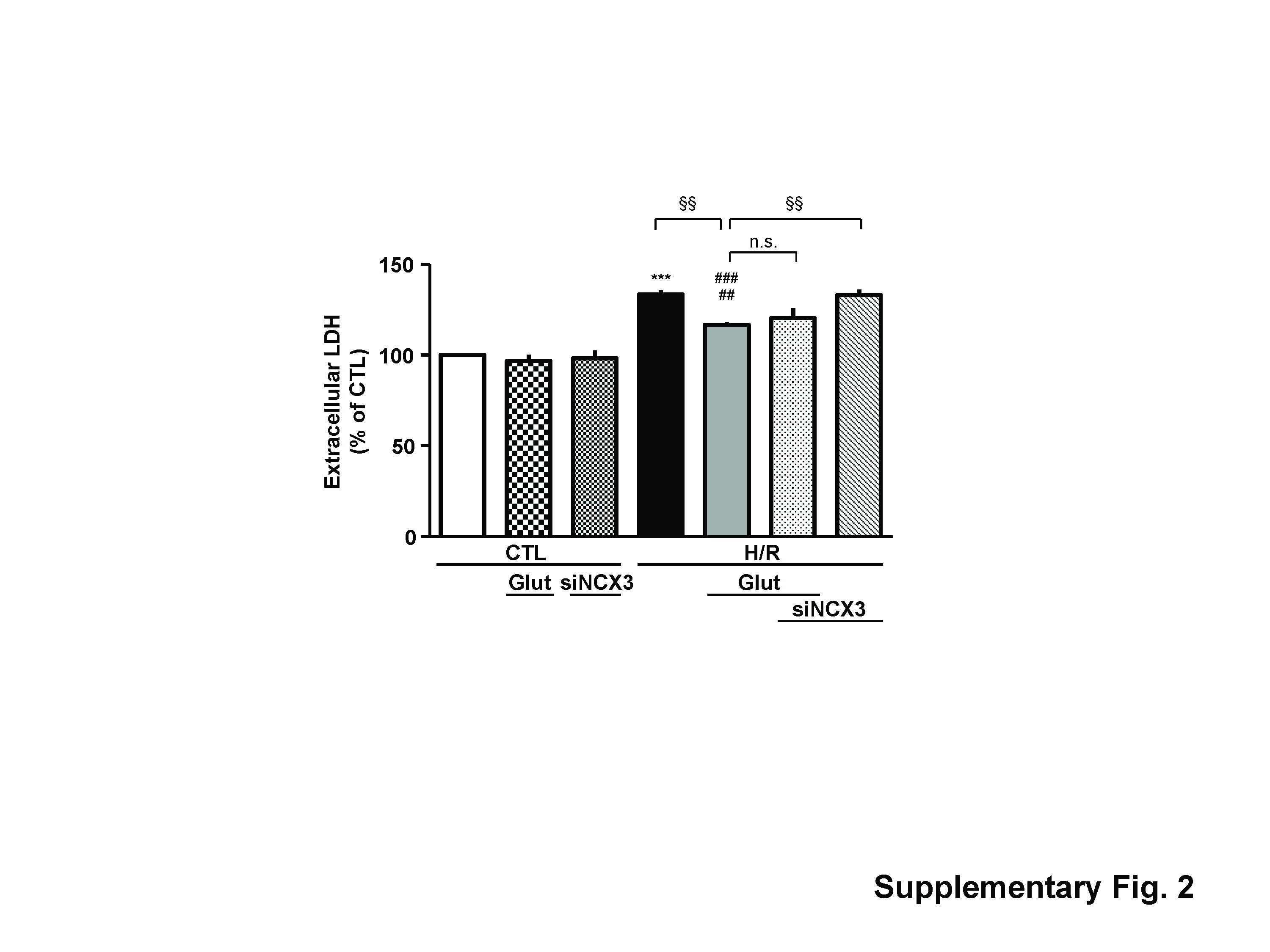

Supplement: Supplementary file 2 — Supplementary Fig. 2 [file 41419_2018_784_MOESM2_ESM.jpg]
