# Supplementary material for: Glutamate as a potential “survival factor” in an in vitro model of neuronal hypoxia/reoxygenation injury: leading role of the Na+/Ca2+ exchanger
Source: Cell Death Dis. 2018 Jun 28;9(7):731. doi: 10.1038/s41419-018-0784-6 (PMC6023866; doi:10.1038/s41419-018-0784-6)
Supplement: Supplementary file 3 — Supplementary information [file 41419_2018_784_MOESM3_ESM.docx]

**Supplementary information**

**Figure legends**

**Figure S1.** Silencing of NCX1 (a), NCX3 (b) and EAAC1 (c) was performed by using HyPerfect Transfection® Kit (Qiagen) and FlexiTube siRNA as described in the “Materials and Methods” section.

CTL = control; sham = siRNA negative control; siNCX1 = siRNA for NCX1; siNCX3 = siRNA for NCX3;

siEAAT3 = siRNA for EAAT3. *p<0.05 vs sham.

**Figure S2.** Effect of NCX3 silencing on glutamate-induced protection against H/R injury. Extracellular LDH activity measured at the end of the H/R protocol (16 h of hypoxia followed by 24 h of reoxygenation) under different experimental conditions. In each experiment, LDH release was expressed as percentage of the control . Silencing of NCX3 was performed 30 h before H/R challenge. Glutamate was added at the onset of the reoxygenation phase. Each column represents the mean ± S.E.M. of almost 5 independent experiments performed in triplicate. Differences among means were assessed by one-way ANOVA followed by Dunnet’s *post hoc* test. F(6, 32) = 23.45. ***p<0.001 vs control groups; ###p<0.0001 vs CTL+Glut; ##p<0.001 vs CTL and CTL+siNCX3; §§p<0.001vs the indicated groups. The H/R group was not significantly different from the HR+siNCX3 group.

There was no statistically significant difference between the control groups.

CTL = control; H/R = hypoxia/reoxygenation; Glut = glutamate; siNCX3 = siRNA for NCX3; n.s. = not significant.
